# A Purified Anthraquinone-Glycoside Preparation From Rhubarb Ameliorates Type 2 Diabetes Mellitus by Modulating the Gut Microbiota and Reducing Inflammation

**DOI:** 10.3389/fmicb.2019.01423

**Published:** 2019-06-25

**Authors:** Hong-Xin Cui, Ling-Shuai Zhang, Yang Luo, Ke Yuan, Zhi-Yong Huang, Ying Guo

**Affiliations:** ^1^College of Pharmacy, Henan University of Chinese Medicine, Zhengzhou, China; ^2^Collaborative Innovation Center for Respiratory Disease Diagnosis and Treatment & Chinese Medicine Development of Henan Province, Zhengzhou, China; ^3^Jiyang College of Zhejiang Agriculture and Forestry University, Zhuji, China; ^4^Zhejiang Chinese Medical University, Hangzhou, China

**Keywords:** anthraquinone-glucoside, diabetes, gut microbiota, intestinal integrity, inflammation

## Abstract

*Rheum palmatum* L. is widely used in traditional Chinese medicine for the treatment of constipation. Here, the therapeutic effects and underlying mechanisms of purified anthraquinone-glycoside preparation from rhubarb (RAGP) on the type 2 diabetes mellitus (T2DM) rats were investigated. After 6 weeks of metformin and RAGP treatment, the weight returned to normal. Fasting blood glucose (FBG), glycated serum protein (GSP), insulin concentration and HOMA-IR index had significantly decreased, and glucagon-like peptide-1 (GLP-1) concentrations had increased. Histological abnormalities in the pancreas and ileum had improved. These effects were associated with enhanced intestinal integrity, thereby reducing the absorption of lipopolysaccharide (LPS) and inflammation. To investigate whether RAGP ameliorated insulin resistance *via* effects on the gut microbiota, we performed 16s rDNA sequencing of ileal gut contents. This showed an amelioration of gut dysbiosis, with greater abundance of probiotic *Lactobacillus* and short-chain fatty acid-producing bacteria, and lower abundance of the Lachnospiraceae NK4A136 group and LPS-producing *Desulfovibrio*. The mechanism of the hypoglycemic effect of RAGP involves regulation of the gut microbiota, activation of the GLP-1/cAMP pathway to ameliorate insulin resistance. Thus, this study provides a theoretical basis for the use of RAGP to treat T2DM, and it may be a novel approach to restore the gut microbiota.

## Introduction

*Rheum palmatum* L., a perennial herb belonging to the Polygonaceae, is well-known in TCM for use in patients with constipation or gastrointestinal hemorrhage and ulcers, and it has antibacterial, anti-inflammatory properties (Zargar et al., 2011). Rhubarb contains a variety of potentially bioactive components, including anthraquinones, bianthrone, stilbenes, polysaccharides, and tannins. In many plants, anthraquinones mainly exist in a combined form (Cao and Zhou, 2009). Previous studies have shown that the total anthraquinone content of rhubarb is ∼4.5% (w/w), of which free anthraquinones account for 1.9% (w/w), while the remainder are present as glycosides (Arvindekar et al., 2015).

Type 2 diabetes mellitus (T2DM) is widespread worldwide, with an annually escalating incidence. According to statistics from the International Diabetes Federation demonstrate, there were 415 million diabetic patients worldwide in 2015, and this number will increase to 642 million by 2040 (Rocha Fernandes et al., 2016). T2DM represents a significant threat to health, but it has complex pathogenesis. T2DM involves multiple disorders, including of lipid and glucose metabolism, β-cell dysfunction, chronic low-grade inflammation, and oxidative stress, which result in insulin resistance and insufficient insulin secretion (Tangvarasittichai, 2015; Gutierrez-Rodelo et al., 2017). Rhubarb and other Chinese herbal decoctions have been used to treat diabetes and its complications (Li et al., 2004; Aditya et al., 2015), but anti-diabetic effects of anthraquinone-glycosides have rarely been reported and its mechanism are still unclear.

Recent studies have shown that gut dysbiosis is also a key underlying defect in T2DM (Han and Lin, 2014; Patterson et al., 2016). Approximatively 1–2 kg of bacteria resides in the human body, 90% of which live in the intestine (Wang, 2017). The balance of the gut microbiota is of great significance for the maintenance of appropriate whole-body metabolism (Jiang, 2014). Furthermore, there is now a large volume of literature to show that imbalance in the gut microbiome is associated with diseases, including obesity, T2DM, and liver cirrhosis (Qin et al., 2012; Karlsson et al., 2013; Qin et al., 2014). The gut microbiota and their metabolites can influence energy balance and glucose metabolism, and induce low-grade inflammation, all of which are important factors for the development of T2DM (Chassaing and Gewirtz, 2014; Mikkelsen et al., 2015). However, previous paper have reported that dietary fiber intake can increase the abundance of SCFA-producing bacteria in the intestine, which can ameliorate T2DM (Zhao L.P. et al., 2018). SCFAs are a stimulus for the secretion of glucagon-likepeptide-1 (GLP-1), the action of which is a target for the treatment of T2DM. Among the TCMs that have been investigated for their effects in T2DM, xiexin tang has been shown to ameliorate T2DM in rats by modulating the gut microbiota (Wei et al., 2018). In addition, emodin has been shown to ameliorate chronic kidney disease by reducing the number of harmful bacteria and altering the gut microbial composition (Zeng et al., 2016). Anthraquinone glycosides have been shown to be mainly absorbed in the intestine and have a bacteriostatic effect as well as anti-inflammatory effects (Zargar et al., 2011; Luo et al., 2013). Yu et al. (2018) also found that the antibacterial activity of anthraquinone-glycoside against pathogenic bacteria is stronger than that of probiotics by culturing several probiotics and pathogenic bacteria *in vitro*. It is unclear whether the hypoglycemic mechanism of anthraquinone-glycosides is related to the regulation of gut microbiota.

Therefore, we aimed to determine whether purified anthraquinone-glycoside preparation from rhubarb (RAGP) can ameliorate T2DM by modulating the gut microbiota and/or having an anti-inflammation effect.

## Materials and Methods

### Materials

The dried rhizomes of *R. palmatum* were obtained from Zhejiang Chinese Medicine University Medicine Co., Ltd. (Zhejiang, China). Diaion HP-20 macroporous resin was purchased from Mitsubishi Group (Tokyo, Japan). Emodin standard and STZ was purchased from Aladdin Bio-Reagents (Shanghai, China). Metformin was purchased from China Associate Pharmaceutical Co., Ltd. (Shenzhen, China). Glucose, GSP, insulin, and GLP-1 kits were purchased from Nanjing Jiancheng Biology Technology Co., Ltd. (Jiangsu, China). A Mag-MK Soil Genome DNA Extraction kit was purchased from Sangon Biotech Co., Ltd., (Shanghai, China). Antibodies against occludin, ZO-1 and β-actin were purchased from Wanlei Biology Technology Co., Ltd. (Liaoning, China). All the reagents were of analytical or HPLC grade.

### Preparation of RAGP

Three kilograms of rhubarb powder was reflux-extracted with six times the volume of 80% ethanol three times for 1.5 h each. The combined filtrate was then concentrated and the ethanol removed using a reduced pressure evaporator. The extractum was then mixed 1:1 in distilled water by ultrasonication. Following this, the solution was extracted with petroleum ether, ethyl acetate, and *n*-butanol, in sequence, with the *n*-butanol fraction containing the anthraquinone glycosides. This crude extract was purified by chromatography using a Diaion HP-20 column, and successively eluted with three times the column volume of distilled water, 10% MeOH, 20% MeOH, 40% MeOH, 60% MeOH, and 80% MeOH. The eluates were collected separately and analyzed by thin layer chromatography. The 40 and 60% MeOH eluates were combined and dried using the reduced pressure evaporator to obtain the purified total anthraquinone glycoside extract. This extract was separated and the components identified as EMG, AEG, and CPG by physicochemical and spectroscopic analysis in our laboratory, and calibrated successively to purities of 98.56, 98.13, and 99.32% using the HPLC peak area normalization method. The total anthraquinone glycoside content was then determined by UV spectrophotometry using EMG as a standard.

The anthraquinone glycoside (EMG, AEG, and CPG) content was determined by HPLC. Chromatographic analysis was undertaken using an Agilent Extend-C18 (4.6 mm × 250 mm, 5 μm) column and a mobile phase of acetonitrile: 0.1% formic acid (90:10, v/v). The flow rate was 1.0 mL/min, the detection wavelength was 267 nm, the column temperature was 30°C, and 20 μL of sample was used.

### Animal Experiments

Male Sprague-Dawley rats (100–120 g) were acclimated to the experimental conditions of 20 ± 2°C, humidity 60 ± 5%, 12-h light/dark cycle, and *ad libitum* food and water, for 1 week. Rats and food were purchased from the Laboratory Animal Center of Zhejiang Academy of Medical Sciences (Zhejiang, China; Certificate Number SCXK 2014-0001). All animal experimentation procedures were conducted in accordance with the Chinese Guidelines for Animal Care, which conform with the internationally accepted uses of experimental animals.

T2DM was induced by consumption of a high fat-high sucrose diet, containing 20% lard, 20% sucrose, and 2.5% cholesterol, for 12 weeks, followed by an intraperitoneal (i.p.) injection of a single low dose of STZ dissolved in normal saline (35 mg/kg) (Li et al., 2017). Rats in the control group received an equivalent volume of normal saline. T2DM was confirmed by retro-orbital blood sampling 48 h after STZ injection for the measurement of FBG. An FBG >7.0 mmol/L was taken to indicate diabetes, and the rat was included in the study. All the included rats were randomly divided into five groups (*n* = 6 per group): normoglycemic rats administered distilled water (NC); T2DM rats administered distilled water (T2DM); T2DM rats administered metformin (MET, 100 mg/kg); T2DM rats administered a LOW (100 mg/kg); and T2DM rats administered a HIG (400 mg/kg). Each rat was gavaged once daily for 6 weeks. This dose administered to T2DM rats has proven to be safe and effective by preliminary testing. During this time, all rats were allowed free access to food and water, and their body masses were recorded weekly.

### Biochemical Analyses

After 6 weeks of RAGP, metformin, or water administration, the rats were terminally anesthetized using 3.5% chloral hydrate, i.p. Blood samples were collected from the abdominal aorta and centrifuged at 3,500 ×*g* for 20 min at 4°C for serum collection. Serum FBG, GSP, insulin, and GLP-1 were measured according to the instructions of the commercially available kits, and then measured on a microplate reader (Multiskan FC, Thermo Fisher Scientific, Waltham, MA, United States). The HOMA-IR was calculated for each rat using the following equation:

HOMA−IR=[FBG (mmol/L)×fasting insulin (mlU/L)]/22.5.

### Histological Examination

Pancreas and ileum were dissected and then washed with phosphate-buffered saline. The tissues were fixed with 4% paraformaldehyde for 24 h, embedded in paraffin, cut into 5-μm-thick sections, stained with H&E, and examined using a light microscope (BX20, Olympus, Tokyo, Japan).

### Western Blotting

Portions of ileum were lysed with protease inhibitor, and the protein content of each quantified using a bicinchoninic acid protein assay kit. Equal amounts of protein (50 μg per lane) were resolved on 12% polyacrylamide gels and transferred to polyvinylidene fluoride membranes (Millipore, Marlborough, MA, United States). After blocking with 4% skim milk for 3 h, membranes were incubated overnight with specific primary antibodies against occludin, ZO-1 or β-actin at room temperature. Each membrane was subsequently washed three times with tris-buffered saline-Tween-20 for 5 min and then incubated with horseradish peroxidase-conjugated secondary antibody for 2 h at 4°C. The proteins were then visualized using an enhanced chemiluminescence detection system (Amersham Pharmacia, Piscataway, NJ, United States).

### Gut Microbiome Analysis

The total fecal genome was extracted under aseptic conditions using a Mag-MK Soil Genome DNA Extraction kit. PCR amplification of the V3–V4 region of 16S rDNA was then performed, and then the purified amplicons were analyzed using paired-end sequencing on the Illumina MiSeq platform by GENEWIZ Technology Inc software (Jiangsu, China), and the raw sequences were processed and analyzed using the Quantitative Insights into Microbial Ecology (QIIME; Version 1.9.1) software package. All sequences were clustered into operational taxonomic units (OTUs) according to 97% similarity, principal component analysis (PCA) was conducted, the alpha diversity index was calculated, and the relative abundance of each bacterial taxon was analyzed by comparison with Silva128 16S rRNA database and QIIME.

### Statistical Analysis

Data were analyzed by one-way ANOVA using SPSS software (Version 16.0) and are expressed as mean ± SD. Significant treatment differences were identified using Tukey’s multiple comparison test. Spearman’s correlation analyses were performed to identify relationships between variables. *P* < 0.05 was considered to represent statistical significance.

## Results and Discussion

### Quantification of Active Ingredients in the Purified RAGP

**FIGURE 1:**
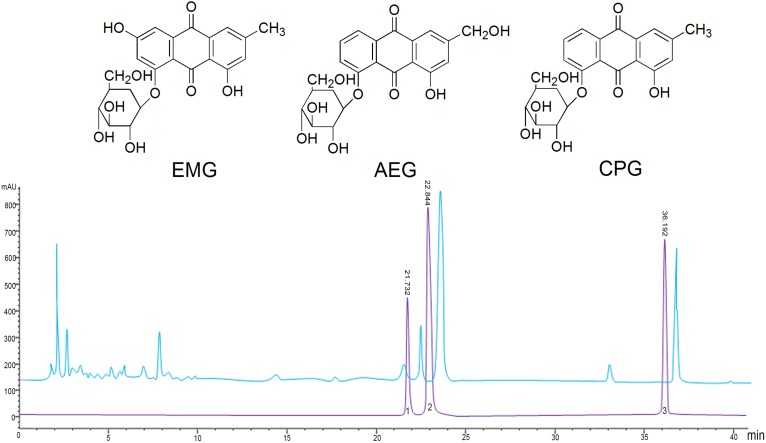
The structure and HPLC chromatography of three anthraquinone glycosides in RAGP. 1: Emodin-8-*O*-β-D-glucoside (EMG), 2: Aloe-emodin-8-*O*-β- D-glucoside (AEG), 3: Chrysophanol-8-*O*-β-D-glucoside (CPG).

It is recognized that the active ingredients of rhubarb are mainly anthraquinones, which exist in free or glycosidated forms in plants (Zargar et al., 2011). The total anthraquinone glycoside content was determined to be 58% in RAGP. The content of EMG, AEG, and CPG in RAGP were 2.05, 6.61, and 3.73%, respectively, as determined by HPLC (Figure 1).

### Effects of RAGP on Body Mass and Serum Biochemistry in T2DM Rats

**FIGURE 2:**
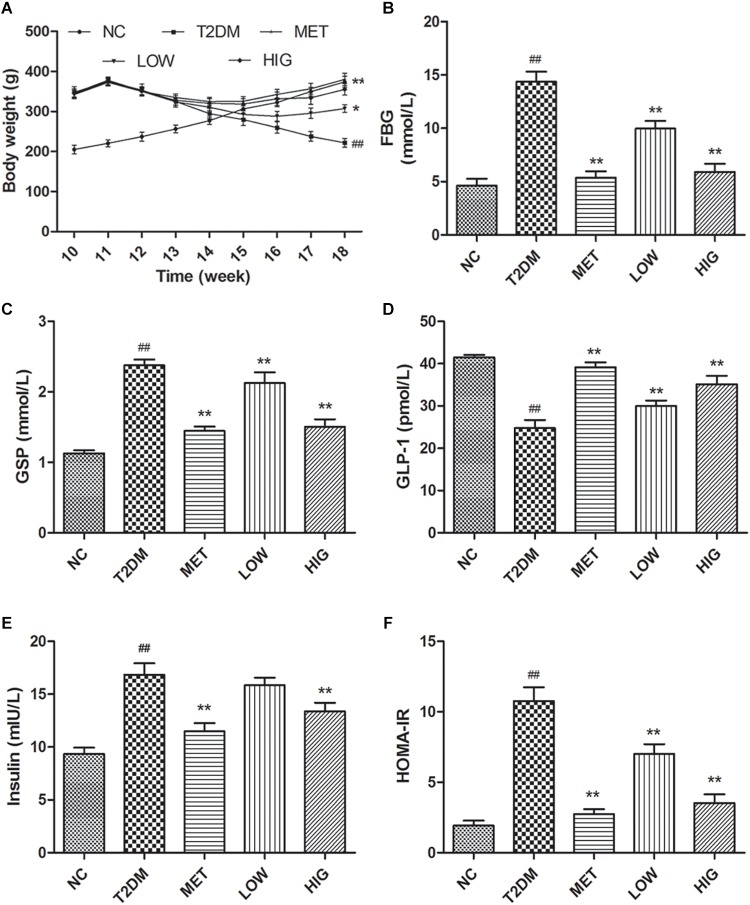
Effect of RAGP on body mass and serum biochemistry in each group (*n* = 6). **(A)** Body weight of all rats from 10 to 18 weeks. At 12th week, T2DM rats were injected with STZ (i.p.). **(B)** Serum FBG, **(C)** GSP, **(D)** serum GLP-1, **(E)** fasting serum insulin, and **(F)** HOMA-IR were tested at the end of the 18 weeks. Values are mean ± SD, ^#^*P* < 0.05, ^##^*P* < 0.01 vs. NC group; ^∗^*P* < 0.05, ^∗∗^*P* < 0.01 vs. T2DM group.

In this study, we used a high-fat, high-sugar diet and low-dose STZ i.p. injection to induce diabetes in rats that mimics the characteristics of human T2DM. T2DM rats exhibited polydipsia, polyphagia, polyuria, emaciation, and weakness by observation. To monitor the progression of T2DM and the effects of therapy, the rats were weighed weekly throughout the experiment. The rats fed high-fat, high-sugar diets for 12 weeks had higher body mass than those in NC group, but after injection of STZ (week 12), this decreased rapidly in T2DM rats, while continuing to increase in the NC group. From weeks 13 to 18, the rats in NC group demonstrated a normal mental state and gained body mass steadily, while rats in T2DM group demonstrated an impaired mental state and decreasing body mass (*P* < 0.05). However, the T2DM rats in MET, LOW, and HIG groups slowly regained body mass, and high-dose RAGP treatment had a greater effect (Figure 2A, *P* < 0.05).

Abnormal glucose metabolism and insufficient insulin secretion are both features of diabetes (Azofeifa et al., 2016). Long-term high-fat diet-feeding and STZ administration causes both pancreatic β-cell damage and insulin resistance (Zhang et al., 2018). At the end of week 12, the FBG and GSP concentrations in T2DM rats were >7.0 and 2.2 mmol/L, respectively, indicating that T2DM rats were induced successfully. The GSP concentration reflects the mean blood glucose concentration over the preceding 2–3 weeks, which is useful for the diagnosis and monitoring of diabetic patients (Chen, 2011). At the end of week 18, the FBG and GSP concentrations in untreated T2DM rats were significantly higher than those of the NC group (*P* < 0.01). However, these were much lower in the MET, LOW, and HIG groups than in the T2DM group (Figure 2B,C, *P* < 0.01). Metformin is one of the most commonly used oral hypoglycemic agents in the clinic, and its hypoglycemic mechanism is mainly through reducing liver gluconeogenesis and inhibiting the intestinal absorption of sugar (Ferrucci and Fabbri, 2018). Therefore, the hypoglycemic effect of metformin was stronger than that of RAGP, but their hypoglycemic mechanism was different.

GLP-1 is a peptide hormone coded by the proglucagon gene and secreted by intestinal epithelial L cells, the action of which is a target of a number of drugs for T2DM (Kuhre et al., 2015). GLP-1 has been shown to increase insulin sensitivity, stimulate insulin secretion, reduce islet β cell apoptosis, promote β-cell proliferation, protect islet cells from glucose toxicity and inflammatory damage, and to act on the central nervous system (especially the hypothalamus) to induce a feeling of satiety (Guo et al., 2015). Therefore, the serum GLP-1 concentration is also important in T2DM. As shown in Figure 2D, GLP-1 concentration was significantly decreased in T2DM group compared with NC group, and metformin and RAGP significantly promoted the secretion of GLP-1 in T2DM rats (*P* < 0.01). GLP-1 binds to its receptors on islet β-cells, increasing the intracellular concentration of cAMP. The consequent activation of protein kinase A (PKA) stimulates insulin secretion and promotes β-cell growth (Purves et al., 2009; Holst et al., 2011). Serum insulin and HOMA-IR was significantly higher in the T2DM group than in the NC group, but that metformin and 400 mg/kg RAGP treatment significantly decreased serum insulin and HOMA-IR (Figure 2E,F, *P* < 0.01). The serum insulin in LOW group did not demonstrate a significant difference, compared with T2DM group. These results imply that RAGP alleviates insulin resistance and hyperglycemia, justifying its use for the treatment of T2DM and its complications.

### Effects of RAGP on Pancreatic and Ileum Tissue Histology

**FIGURE 3:**
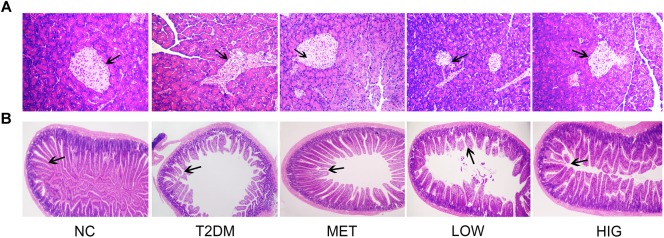
Effects of RAGP on histopathological analysis. **(A)** Pancreas tissues stained with H&E (100×); **(B)** ileum tissues stained with H&E (25×).

The hyperglycemia observed in the T2DM mice is likely to be associated with the production a large quantity of superoxide, which induces pancreatic and ileum damage (Giugliano et al., 1996). Some anthraquinone compounds have been reported to have antioxidant effects (Zargar et al., 2011; Wang et al., 2016); therefore pancreatic histology was analyzed following H&E staining of tissue sections (Figure 3A). The islets of rats in the NC group were round or oval, with well-defined boundaries, and were filled with dense, well-distributed endocrine cells. By contrast, the islets of rats in the T2DM group were irregularly shaped, with unclear margins, nuclear pyknosis, vacuolar degeneration, inflammatory cell infiltration, and fewer β-cells. However, these features were less marked and the number of islet β-cells was significantly higher in rats in the MET and RAGP-treated groups than in the T2DM group.

Gastrointestinal dysfunction is a common complication in T2DM patients, manifesting for example as abnormal intestinal emptying, diarrhea, or constipation (Yang et al., 2015). H&E-stained ileum sections from rats in the NC group displayed intact architecture with well-arranged villi and crypts (Figure 3B), whereas sections in T2DM group demonstrated marked abnormalities, including abnormal intestinal walls, disorganized and collapsed villi, and swollen and degenerate villus epithelium. However, MET, LOW, or HIG groups were associated with less intestinal pathology, and this effect was more pronounced in the HIG group. These findings are in agreement with those of a previous study (Zhang et al., 2017).

### Effects of RAGP on Intestinal Integrity

**FIGURE 4:**
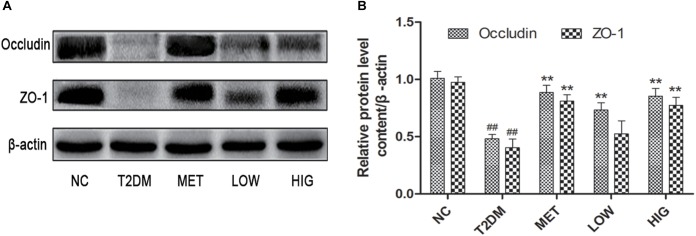
Effects of RAGP on the expression level of ZO-1 and occludin in the ileum tissues in each group (*n* = 6). **(A)** The expression levels of ZO-1 and occludin were analyzed by western blotting. **(B)** The density identification of occluding, ZO-1 were calculated as shown. Values are mean ± SD, ^#^*P* < 0.05, ^##^*P* < 0.01 vs. NC group; ^∗^*P* < 0.05, ^∗∗^*P* < 0.01 vs. T2DM group.

The chronic low levels of inflammation present in the gut of diabetic patients are associated with the degradation of intestinal tight junctions (Fava, 2014). ZO-1 and occludin are major tight junction proteins found in the intestine; if these are present at lower concentrations, greater intestinal permeability results (Sato et al., 2017). Previous studies have found that the small intestinal integrity of diabetic patients is impaired because of the thinning of the intestinal mucus layer, a lack of particular antimicrobial peptides and tight junction proteins, and oxidative stress (Brun et al., 2007; Cani et al., 2009). As shown in Figure 4A, the protein expression of ZO-1 and occludin was significantly lower in T2DM group. However, treatment with metformin or high-dose RAGP significantly increased the expression of ZO-1 and occludin (Figure 4B, *P* < 0.01), but low-dose RAGP had no obvious effect on ZO-1. Low tight junction protein expression in the intestine of diabetic patients increases intestinal permeability, such that the lipopolysaccharide (LPS) produced by Gram-negative bacteria can be absorbed in larger quantities, causing chronic inflammation by activating the NF-κB signaling pathway (Cani et al., 2009). It has been also shown that emodin reduces inflammation in rats with acute pancreatitis (Ma et al., 2005). Moreover, RAGP in intestine can be decomposed and inhibit the activity of Na^+^-K^+^-ATPase, which promotes intestinal water secretion (Xie et al., 2018). Hattori et al. (1982) and Song et al. (2011) also found that anthraquinone-glycosides entering into the intestinal tract are mainly decomposed into a glycone by enzymes (β-glucosidase) secreted by the intestinal flora, thereby exerting antibacterial and anti-inflammatory action. Therefore, it may promote the growth of probiotics and inhibit the production of LPS, thereby upregulating the expression of tight junction proteins and increasing intestinal barrier function. The RAGP-induced improvement in intestinal integrity should therefore reduce this LPS-induced inflammation (Figure 7). Our findings are consistent with those of a previous study that found that RAGP can inhibit inflammatory responses by protecting intestinal tight junctions (Zheng et al., 2018).

### Effects of RAGP on Gut Microbiome Composition in T2DM Rats

The secretion of GLP-1 closely correlates with the quantity of SCFA produced by gut microbial metabolism (Everard and Cani, 2013; Park et al., 2015). Increasing the abundance of SCFA-producing bacteria is another potential approach to the treatment of T2DM. To explore the effects of RAGP on the gut microbiome, Illumina MiSeq sequencing of the V3–V4 region of 16S rDNA was performed. A total of 1,981,228 raw sequences were generated, from which low-quality sequences were removed, leaving 88.1% of the total. The remaining clean tags were clustered into OTUs on the basis of 97% similarity.

**FIGURE 5:**
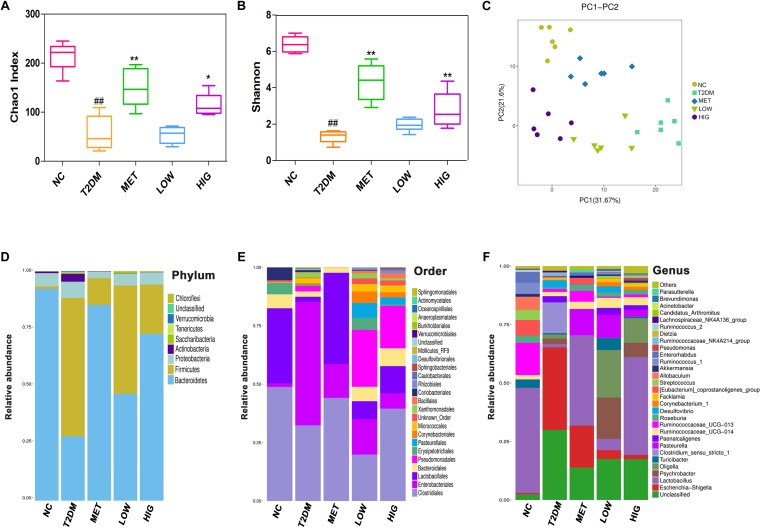
Effect of RAGP treatment on gut microbiome composition of rats in each group (*n* = 6). **(A)** Bacterial richness, **(B)** diversity index, **(C)** principal component analysis (PCA) graphs and microbial community changes at **(D)** phylum level, **(E)** order level, and **(F)** genus level were obtained from ileum fecal samples and analyzed using QIIME. Values are mean ± SD, ^#^*P* < 0.05, ^##^*P* < 0.01 vs. NC group; ^∗^*P* < 0.05, ^∗∗^*P* < 0.01vs. T2DM group.

The human body carries a huge and varied microbiota, in total ∼10 times the number of human cells (Zhang, 2017). Diversity of its microbiota is thought to indicate a “healthy gut” (Sommer et al., 2017). α-Diversity analysis of the intestinal contents showed that the Chao 1 and Shannon values for the T2DM group were significantly lower than those for the NC group, indicating that the abundance and diversity of the gut microbiome in T2DM rats was lower (*P* < 0.01), but these values were higher in MET and HIG groups than in T2DM group (Figure 5A,B, *P* < 0.05). These results are consistent with those of Zhang et al. (2018). Greater microbial diversity is associated with a more robust response to environmental insults, because functionally similar microbes in an intact ecosystem can compensate for the function of other missing species (Sommer et al., 2017). To analyze the β-diversity of the gut microbiome among the rat groups, PCA was conducted, which clusters the gut microbial taxa for each group (Figure 5C).

At the phylum level, the gut microbiome consists largely of Firmicutes, Bacteroidetes, Proteobacteria, Actinobacteria, and Verrucomicrobia, of which the Firmicutes and Bacteroidetes account for 98% of the total number (Wu et al., 2010). Mahowald et al. (2009) found that Bacteroidetes is the only phylum that encodes carbohydrate-degrading enzymes in the human gut microbiome. Frank et al. (2014) transplanted feces from obese rats with a high proportion of Firmicutes and Bacteroidetes into sterile mice, which successfully replicated the obese phenotype. This implies that a change in the abundance of Firmicutes and Bacteroidetes can induce the development of obesity and diabetes. In our study, the gut microbiome of the rats was mainly composed of Bacteroidetes and Firmicutes, and greater abundance of Firmicutes and lower abundance of Bacteroidetes characterized the T2DM group when compared with the NC group, while metformin and RAGP treatment significantly reduced these differences (Figure 5D). Rhein inhibits the activity of Firmicutes and promotes the degradation of carbohydrates by Bacteroidetes (Wang et al., 2016). Zhao C. et al. (2018) also found that the abundance of Bacteroidetes is significantly lower in diabetic animals, but is increased by treatment with an extract of the brown seaweed, *Lessonia nigrescens*.

At the taxonomic order level, the abundance of Clostridiales and Lactobacillales was greater in the MET and HIG groups than in the T2DM group, whereas the abundance of Enterobacteriales was lower in the MET and HIG groups (Figure 5E).

**FIGURE 6:**
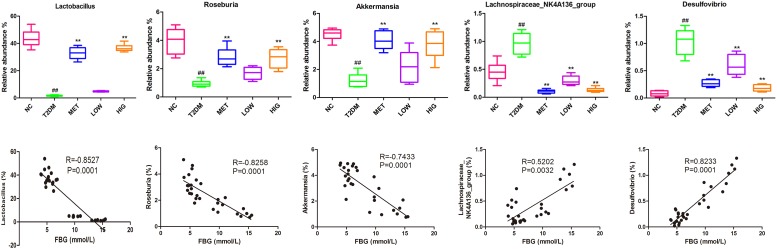
Effect of RAGP treatment on the relative abundance (%) of gut microbial community at genus level and the correlation analysis between relative abundance of gut bacteria and FBG, including Lactobacillus, Roseburia, Akkermansia, Lachnospiraceae NK4A136 group, and Desulfovibrio. The relative abundance of gut microbial community was analyzed using QIIME and Tukey’s multiple comparison test. Values are mean ± SD, ^#^*P* < 0.05, ^##^*P* < 0.01vs. NC group; ^∗^*P* < 0.05, ^∗∗^*P* < 0.01 vs. T2DM group.

At the genus level, some probiotic and SCFA-producing bacteria are associated with diabetes resistance (Patterson et al., 2016). SCFA-producing bacteria can metabolize polysaccharides, such as insoluble starch and indigestible fructose, into SCFAs, including acetate, propionate, and butyrate. These SCFAs act as energy substrates and bind to G-protein-coupled receptor 43/41(GPCR 43/41), causing secretion of additional GLP-1, which ameliorates insulin resistance and lowers blood glucose (Holst et al., 2011; Park et al., 2015). As shown in Figure 5F, 6, significantly fewer *Lactobacillus*, *Roseburia*, and *Akkermansia* were present in T2DM rats, but their numbers were significantly higher in the MET and HIG than those in the T2DM group (*P* < 0.01). In contrast, the abundances of *Desulfovibrio* and Lachnospiraceae NK4A136 were much higher in T2DM rats than in NC rats, but their abundances were markedly decreased in MET and RAGP-treated groups compared with T2DM group (*P* < 0.01). The results indicate that RAGP has a regulatory effect on the growth of probiotics and pathogenic bacteria in the intestine. *Lactobacillus*, a major probiotic, produces lactic acid, CO_2_, acetic acid, and/or ethanol, which contribute to a more acidic environment through homo- or heterofermentative metabolism (Macfarlane and Macfarlane, 1997; Nobaek et al., 2000). This is a beneficial property that makes this genus for the health of the intestinal tract. Our results were consistent with those of previous studies in which it was shown that *Lactobacillus* abundance was lower in diabetic and obese rats but greater in diabetic rats that were under treatment (Yan et al., 2016; Zhang et al., 2018). *Roseburia* is a butyric acid-producing genus that plays a key role in regulating glycolipid metabolism (Udayappan et al., 2014). Butyrate has been shown to induce the secretion of GLP-1, which in turn stimulates insulin secretion (Everard and Cani, 2013). Qin et al. (2012) found that the gut microbiome is disordered in T2DM patients, with a smaller population of certain butyrate-producing bacteria and larger numbers of opportunistic pathogens. *Akkermansia* is a dominant bacterial genus that colonizes the intestinal mucosa, degrades mucin, and produces propionic acid, which provides energy for intestinal epithelial cells, thereby protecting the intestinal mucosal barrier and reducing protein deposition (Collado et al., 2007). Under normal conditions, *Akkermansia* accounts for 3–5% of the gut microbiome and it also protects the intestinal mucosal barrier by reducing the inflammatory response and improving glycolipid metabolism. However, in high-fat diet-fed rodents and diabetic patients, its abundance is lower, whereas it has also been shown that increasing the abundance of *Akkermansia* can ameliorated T2DM (Abbeele et al., 2011; He et al., 2016). The Lachnospiraceae NK4A136 group is an indicator of gut dysbiosis, because when present in great abundance, gut dysbiosis is more severe (Zheng et al., 2018).

Gut dysbiosis in T2DM patients usually involves greater abundance of Gram-negative bacteria. LPS is a component of the outer membrane of Gram-negative bacteria, and their death causes the release of a large quantity of LPS, which can be absorbed into the bloodstream and cause chronic inflammation and insulin resistance (Wang et al., 2018; Wei et al., 2018). *Desulfovibrio* is a Gram-negative genus that can convert sulfate into a toxic gas (H_2_S), which when produced in high concentrations leads to the release of a large amount of LPS, which can destroy the intestinal barrier and increase intestinal permeability (Xiao et al., 2014). Thus, our data suggest that RAGP may ameliorate the disturbance in glucose metabolism by correcting gut microbial imbalance in T2DM rats.

### Correlations Between Metabolic Biomarkers and Bacterial Abundance

To elucidate the relationships between bacterial abundance and FBG, Spearman’s correlation coefficients were calculated. The abundances of *Lactobacillus*, *Roseburia*, and *Akkermansia* were negatively correlated with FBG, while those of *Desulfovibrio* and the Lachnospiraceae NK4A136 group were positively correlated (Figure 6, *P* < 0.01), which is in agreement with previously published findings (Zhang et al., 2018; Zheng et al., 2018).

## Conclusion

**FIGURE 7:**
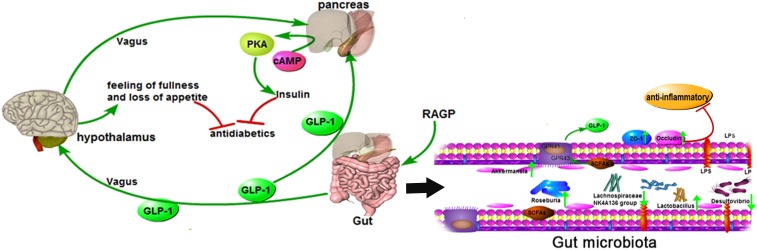
Mechanism of RAGP against T2DM: the interaction of gut microbiota and the reduction of inflammation. RAGP regulated the structure of the gut microbiota, increased the abundance of Lactobacillus and SCFAs-producing (Roseburia and Akkermansia), and decreased Lachnospiraceae NK4A136 group and LPS-producing (Desulfovibrio). Meanwhile, RAGP up-regulated the expression level of ZO-1 and occludin, which improved intestinal integrity and suppressed the LPS-induced inflammation. The improved effect was also indicated by the green arrow after RAGP treatment.

This study demonstrated that RAGP significantly ameliorates T2DM induced by high-fat diet-feeding combined with STZ administration. The mechanism can be attributed to the moderating effect on the gut microbiota. RAGP increased the abundance of certain probiotic and SCFA-producing bacteria, which provide energy for intestinal L cells to secrete more GLP-1. This GLP-1 can then bind to receptors on islet β-cells and increase insulin secretion, as well as improving the sensitivity of peripheral tissues to insulin, thereby ameliorating insulin resistance. GLP-1 also stimulates the satiety center of the hypothalamic to reduce appetite (Figure 7). In addition, RAGP reduced the abundance of LPS-producing bacteria and enhanced intestinal integrity, which would inhibit chronic systemic inflammatory responses. Thus, our findings support the use of RAGP for the treatment of T2DM as a novel approach targeting the correction of gut dysbiosis.

## Data Availability

All datasets generated for this study are included in the manuscript and/or the Supplementary Files.

## Ethics Statement

Rats and their feed were purchased from the Laboratory Animal Center of Zhejiang Academy of Medical Sciences (Zhejiang, China; Certificate Number SCXK 2014-0001). All animal experimentation procedures were conducted in accordance with the Chinese Guidelines for Animal Care, which conform with the internationally accepted uses of experimental animals.

## Author Contributions

H-XC, L-SZ, YL, and KY designed the study. H-XC, L-SZ, YL, Z-YH, KY, and YG conducted the experiments and discussed the results. H-XC, L-SZ, and KY wrote the manuscript.

## Conflict of Interest Statement

The authors declare that the research was conducted in the absence of any commercial or financial relationships that could be construed as a potential conflict of interest.

## Abbreviations

AEG: aloe-emodin-8-*O*-β-D-glycoside
CPG: chrysophanol-8-*O*-β-D-glycoside
EMG: emodin-3-*O*-β-glycoside
FBG: fasting blood glucose
GLP-1: glucagon-like peptide-1
GSP: glycated serum protein
H&E: hematoxylin and eosin
HIG: high dose of RAGP
HOMA-IR: homeostasis model for the assessment of IR
HPLC: high-performance liquid chromatography
LOW: low dose of RAGP
LPS: lipopolysaccharide
MET: metformin
NC: normoglycemic
RAGP: purified anthraquinone glycoside preparation from rhubarb
SCFA: short-chain fatty acid
STZ: streptozotocin
T2DM: type 2 diabetes mellitus
TCM: traditional Chinese medicine
ZO-1: zonula occludens protein-1
